# Concerns About the Generalizability Associated With a South African Randomized Controlled Trial on Prenatal Mothers

**DOI:** 10.2196/53861

**Published:** 2024-02-12

**Authors:** Yongjian Lin

**Affiliations:** 1 Department of Gastrointestinal and Gland Surgery The First Affiliated Hospital of Guangxi Medical University Nanning China

**Keywords:** letter, maternal child health, mHealth, mobile health, randomized controlled trial, short animated storytelling, South Africa, video health messaging

We have carefully reviewed the study conducted by Adam et al [1], which assessed the impact of short animated storytelling (SAS) on prenatal mothers’ maternal knowledge and user satisfaction. The findings of this study indicated that while the SAS videos had high user satisfaction, the measured knowledge gains were limited, which could work as a broad-reaching perinatal health program [1]. In light of our thorough research for this paper, we would like to offer the following suggestions.

First, as the authors described in the Trial Design section, “participants were randomly assigned to either the SAS Intervention group or the Standard-of-Care (SOC) Control group,” rather than using propensity score matching [2]. Most patients have individual polymorphisms in the real world, whereas no significant differences were seen between the SAS intervention group and the control group, which is not consistent with individual polymorphism. Furthermore, when describing data where population characteristics are unknown (eg, knowledge and maternal satisfaction outcomes), a nonparametric test using rank or approximate *t* test may be considered. Therefore, the results of this study may not truly reflect the situation of patients in the real world.

Second, we explored whether the SAS videos played a role in innovative approaches to perinatal health services, and it remains ambiguous whether the relationship between the variables examined in this study (eg, age, education, area, race, and working status) and maternal knowledge was linear or curvilinear. After adjusting for age, sex, education level, living area, and working status, it is advisable to use generalized estimating equations for estimating intervention effects instead of multivariate regression analysis [3]. This is due to the capability of the generalized linear model to account not only for the linear relationship between the two variables but also for the curvilinear relationship between them.

Finally, interaction is important in clinical studies because environmental factors and lifestyle habits may have an impact on clinical outcomes. For example, Yakupova and Liutsko [4] noted that physical health during pregnancy and marital satisfaction significantly predicted prenatal depression based on data from middle-income countries. Another study showed that the climate and physical environment of the care setting consistently influenced women’s satisfaction, despite varying ethnic backgrounds [5]. In this study, many variables were described in Table 1, but subgroup analyses were not conducted based on factors such as age, race, education level, and income. Hence, this paper would be more meaningful if these factors were analyzed in layers.

In conclusion, we thank the authors for this excellent work that provides an approach to perinatal care. However, we believe that the conclusions of this study would change if the previously mentioned issues were further addressed.

## Abbreviations

SAS: short animated storytelling
